# THE SHIFTING SOCIAL NETWORKS OF OLDER ADULTS: AN ANALYSIS OF VARYING TYPOLOGY BY RACE/ETHNICITY

**DOI:** 10.1093/geroni/igac059.546

**Published:** 2022-12-20

**Authors:** Lissette Piedra, James Iveniuk

**Affiliations:** University of Illinois at Urbana-Champaign, Urbana, Illinois, United States; NORC at the University of Chicago, Chicago, Illinois, United States

## Abstract

We explored whether network groupings change over time, and how they vary by race and ethnicity. We draw upon data from 6,489 respondents, collected across three rounds of National Social Life Health and Aging Project (NSHAP). Using Latent Class Analysis to identify groupings of social-relational characteristics, we arrive at: A ‘restricted’ class with overall low social connectivity, an ‘enriched’ class with strong connections across all domains and a ‘diverse’ class showing the greatest network range. In the first round, 49% of Hispanic respondents were in the ‘restricted’ class, compared to 24% of non-Hispanic Whites, and 37% of non-Hispanic Blacks. By the third round, the percentage of Hispanic respondents in the ‘restricted’ class dropped but rose for all other groups. We speculate that, over time, context-specific factors may be contributing to network resilience among Hispanics and network vulnerability among non-Hispanic Blacks.

